# Ethylene-induced potassium transporter *AcKUP2* gene is involved in kiwifruit postharvest ripening

**DOI:** 10.1186/s12870-022-03498-9

**Published:** 2022-03-09

**Authors:** Nan Shan, Yupei Zhang, Yunhe Xu, Xin Yuan, Chunpeng Wan, Chuying Chen, Jinyin Chen, Zengyu Gan

**Affiliations:** 1 Jiangxi Key Laboratory for Postharvest Technology and Nondestructive Testing of Fruits and Vegetables, Collaborative Innovation Center of Postharvest Key Technology and Quality Safety of Fruits and Vegetables, Jiangxi Agricultural University, Nanchang, 330045 China; 2 College of Materials and Chemical Engineering, Pingxiang University, Pingxiang, 330075 China

**Keywords:** Potassium transporter, Fruit ripening, Gene expression, Ethylene, Promoter activity, *Actinidia chinensis*

## Abstract

**Background:**

Potassium (K) is important in the regulation of plant growth and development. It is the most abundant mineral element in kiwifruit, and its content increases during fruit ripening. However, how K^+^ transporter works in kiwifruit postharvest maturation is not yet clear.

**Results:**

Here, 12 K^+^ transporter *KT/HAK/KUP* genes, *AcKUP1* ~ *AcKUP12*, were isolated from kiwifruit, and their phylogeny, genomic structure, chromosomal location, protein properties, conserved motifs and *cis*-acting elements were analysed. Transcription analysis revealed that *AcKUP2* expression increased rapidly and was maintained at a high level during postharvest maturation, consistent with the trend of K content; *AcKUP2* expression was induced by ethylene, suggesting that *AcKUP2* might play a role in ripening. Fluorescence microscopy showed that AcKUP2 is localised in the plasma membrane. *Cis*-elements, including DER or ethylene response element (ERE) responsive to ethylene, were found in the *AcKUP2* promoter sequence, and ethylene significantly enhanced the *AcKUP2* promoter activity. Furthermore, we verified that AcERF15, an ethylene response factor, directly binds to the *AcKUP2* promoter to promote its expression. Thus, *AcKUP2* may be an important potassium transporter gene which involved in ethylene-regulated kiwifruit postharvest ripening.

**Conclusions:**

Therefore, our study establishes the first genome-wide analysis of the kiwifruit *KT/HAK/KUP* gene family and provides valuable information for understanding the function of the *KT/HAK/KUP* genes in kiwifruit postharvest ripening.

**Supplementary Information:**

The online version contains supplementary material available at 10.1186/s12870-022-03498-9.

## Background

Potassium (K) is one of the most abundant mineral elements in plant cells, and it affects the growth and development of the whole plant through its involvement in water metabolism and assimilate transport [1]. Currently, many studies have confirmed that potassium deficiency negatively affects the yield and quality of fruits [2, 3]. Potassium can promote the expansion of grapefruits [4], and, significantly, the synthesis of sucrose and starch in tomato [5], melon [6], strawberry [7], and apple [8]. Potassium is also involved in fruit ripening. For example, potassium content increases as bananas ripen [9]; during grapefruits maturation, K^+^ transport is involved in the unloading of phloem assimilates [4]. Moreover, K^+^ also participates in fruit ripening by affecting soluble sugar accumulation and the acid metabolism pathway [10–12].

K^+^ absorption and release involves the transport of K^+^ across the plasma membrane [13], and K^+^ entry and exit into the vacuole involve transport across the vacuole membrane [14]. The transport of K^+^ depends on many channels or transporters, including three K^+^ channel families (Shaker, TPK, and Kir-like) [1, 15–17] and three K^+^ transporter families (KT/HAK/KUP, HKT, and CHX) [15, 18–20]. KT/HAK/KUP is the earliest discovered and the most prolific in numbers and functions; however, its primary function is to maintain cellular K^+^ homeostasis [21]. The first members of the *KT/HAK/KUP* family identified in plants were *AtKUP1* in *Arabidopsis thaliana* [22] and *HvHAK1* in barley [23]. Subsequently, other members were also identified in different species, such as rice [24], tomato [25], and pear [26]. According to the different degrees of K^+^ affinity, the family can be divided into high-, low-, and dual-affinity K^+^ transporters. *OsHAK5* encodes a high, and *OsHAK7/10* a low-affinity K^+^ transporter [24, 27]. Conversely, *AtKUP1* encodes a high-affinity transporter and also a component with low-affinity absorption [22].

Fruits are a powerful storage of potassium. In this context, they have a large demand for K^+^ from development to full maturity; the transport of K^+^ in fruits is primarily regulated by K^+^ transporters [28]. It has been reported that some K^+^ channel proteins were involved in fruit development, maturation, and quality regulation. *SIRK*, a KAT-type Shaker channel gene, which transcript decreases drastically at veraison, plays a role in the regulation of transpiration and water fluxes in grape [29]. *FaKAT1* and *FaTPK1* perform important roles in fruit ripening and quality formation in strawberry [28, 30]. There have been reports on the expression of the KT/HAK/KUP family during the development and maturation of fleshy fruits [26, 31]; however, their specific involvement and effects on fruit quality are rarely reported.

Kiwifruit is a typical climacteric fruit at room temperature, and ethylene is essential for its ripening [32]. There is a great demand for K^+^ during kiwifruit development and maturity, and K^+^ has a significant effect on the quality and shelf life of kiwifruit after harvest. K^+^ transporters carry K^+^ to the fruit as a movable element during development and maturation. Therefore, in the present study, 12 K^+^ transporter *KT/HAK/KUP* genes were identified from the kiwifruit genome database (KGD) and were subsequently performed a systematic analysis including chromosome location, phylogenetic relationships, gene structure, conserved motif and *cis*-acting elements. We further analyzed the expression of *KT/HAK/KUP* genes during kiwifruit postharvest ripening, and found that *AcKUP2* expression was significantly regulated by ethylene. Furthermore, we verified that AcERF15, an ethylene response factor, directly binds to the *AcKUP2* promoter to promote its expression. This study provides reliable investigation of the *KT/HAK/KUP* gene family in kiwifruit and determined that the *AcKUP2* function in fruit ripening is regulated by ethylene.

## Results

### Analysis of minerals content of kiwifruit pulp at different postharvest stages

The firmness and the content of TSS are two important factors of kiwifruit ripening. The pulp firmness declined from 66.368 to 5.966 N during postharvest ripening, and the TSS of significantly increased along the ripening stage (Table 1). The analysis of minerals revealed that K content was the highest, followed by Mg, Ca, Na and Fe, while Cu content was the lowest. The K content increased along the early stage of fruit ripening and decreased from the eighth day after harvest; firmness also dropped sharply on the eighth day. The content of Ca, Na, and Fe decreased to varying degrees during the postharvest ripening stage, while the content of Mg and Cu have been relatively stable (Table 1).

**Table 1:** Firmness, TSS, and concentration of various minerals at different postharvest stages of kiwifruit pulp

DAP	Firmness (N)	TSS (%)	K (mg/100 g)	Ca (mg/100 g)	Mg (mg/100 g)	Na (mg/100 g)	Fe (mg/100 g)	Cu (mg/100 g)
0	66.368 ± 6.351 a	6.943 ± 0.232 f	170.474 ± 3.511 e	7.687 ± 0.188 a	7.678 ± 0.454 ab	5.438 ± 0.343 b	0.696 ± 0.042 a	0.072 ± 0.006 a
2	60.808 ± 5.706 a	7.512 ± 0.353 e	186.546 ± 4.763 cd	7.332 ± 0.266 ab	7.521 ± 0.353 ab	6.140 ± 0.251 a	0.443 ± 0.029 b	0.060 ± 0.009 a
4	50.752 ± 4.422 b	9.266 ± 0.263 d	194.959 ± 4.231 bc	6.950 ± 0.153 b	7.920 ± 0.417 a	3.749 ± 0.285 c	0.275 ± 0.019 c	0.065 ± 0.010 a
6	32.842 ± 5.798 c	12.843 ± 0.362 c	195.717 ± 3.647 b	6.296 ± 0.275 c	7.702 ± 0.377 ab	3.869 ± 0.153 c	0.227 ± 0.011 d	0.065 ± 0.010 a
8	19.404 ± 3.011 d	14.717 ± 0.183 b	208.064 ± 6.164 a	5.955 ± 0.202 c	7.224 ± 0.189 b	3.674 ± 0.276 c	0.218 ± 0.018 de	0.070 ± 0.012 a
10	8.801 ± 4.602 e	16.320 ± 0.385 a	185.394 ± 3.195 d	4.491 ± 0.193 d	7.505 ± 0.285 ab	3.975 ± 0.346 c	0.185 ± 0.014 e	0.068 ± 0.007 a
12	5.966 ± 3.031 e	16.386 ± 0.293 a	170.419 ± 4.522 e	4.023 ± 0.124 e	7.734 ± 0.214 a	2.173 ± 0.133 d	0.195 ± 0.009 e	0.068 ± 0.006 a

Values represent the mean ± SD. Different letters in the same column indicate that values are statistically different at *P* < 0.05 level

*TSS* total soluble solids, *DAP* days after harvest, *K* potassium, *Ca* calcium, *Mg* magnesium, *Na* sodium, *Fe* iron *Cu* copper

### Identification of *KT/HAK/KUP* genes from kiwifruit

**Fig. 1:**
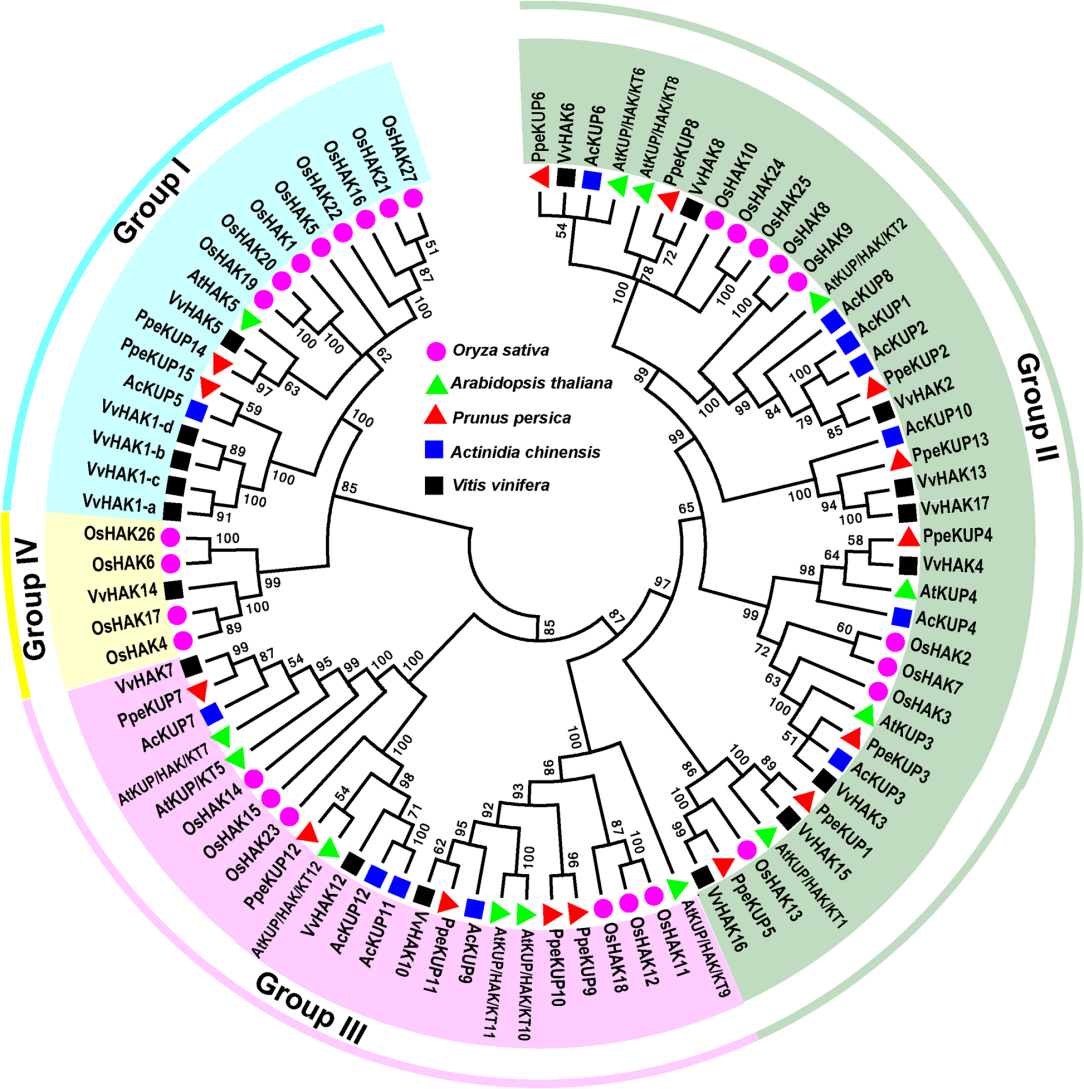
Phylogenetic tree constructed using 85 full-length KT/HAK/KUP proteins from *Oryza sativa*, *Arabidopsis thaliana*, *Prunus persica*, *Actinidia chinensis*, and *Vitis vinifera*. The tree was assembled using the software MEGA 7.0. All the KT/HAK/KUP proteins are divided into four groups (I–IV), and evolutionary history was inferred using the neighbour-joining method with 1,000 replicates. Coloured glyphs represent KT/HAK/KUPs from different species: *O. sativa* (Os; pink circle), *A. thaliana* (At; green triangle), *P. persica* (Ppe; red triangle), *A. chinensis* (Ac; blue square), *V. vinifera* (Vv; black square)

Using sequences of KT/HAK/KUP transporters from *A. thaliana*, candidate KT/HAK/KUP transporters were identified from the KGD. Among them, 12 putative *KT/HAK/KUP* genes (designated *AcKUP1*–*AcKUP12*) were identified. Information about them is listed in Table S1, The AcKUPs ranged from 619 (AcKUP4) to 931 (AcKUP11) amino acid residues in length, corresponding to calculated molecular weights from 69.03 to 103.09 kDa. Estimated isoelectric points ranged from 4.91 (AcKUP7) to 9.43 (AcKUP4). All AcKUPs harboured 6–13 transmembrane helices and were predicted to be located predominantly in the plasma membrane (Table S1). In total, 85 full-length protein sequences from kiwifruit (12), peach (15), grapevine (18), *A. thaliana* (13) and rice (27) were used to construct a phylogenetic tree. As shown in Fig. 1, the HAK/KUP/KTs were s divided into four major groups (I–IV); 12 AcKUP proteins were distributed on groups I–III, with 1, 7, and 4 members, respectively (Fig. 1).

**Fig. 2:**
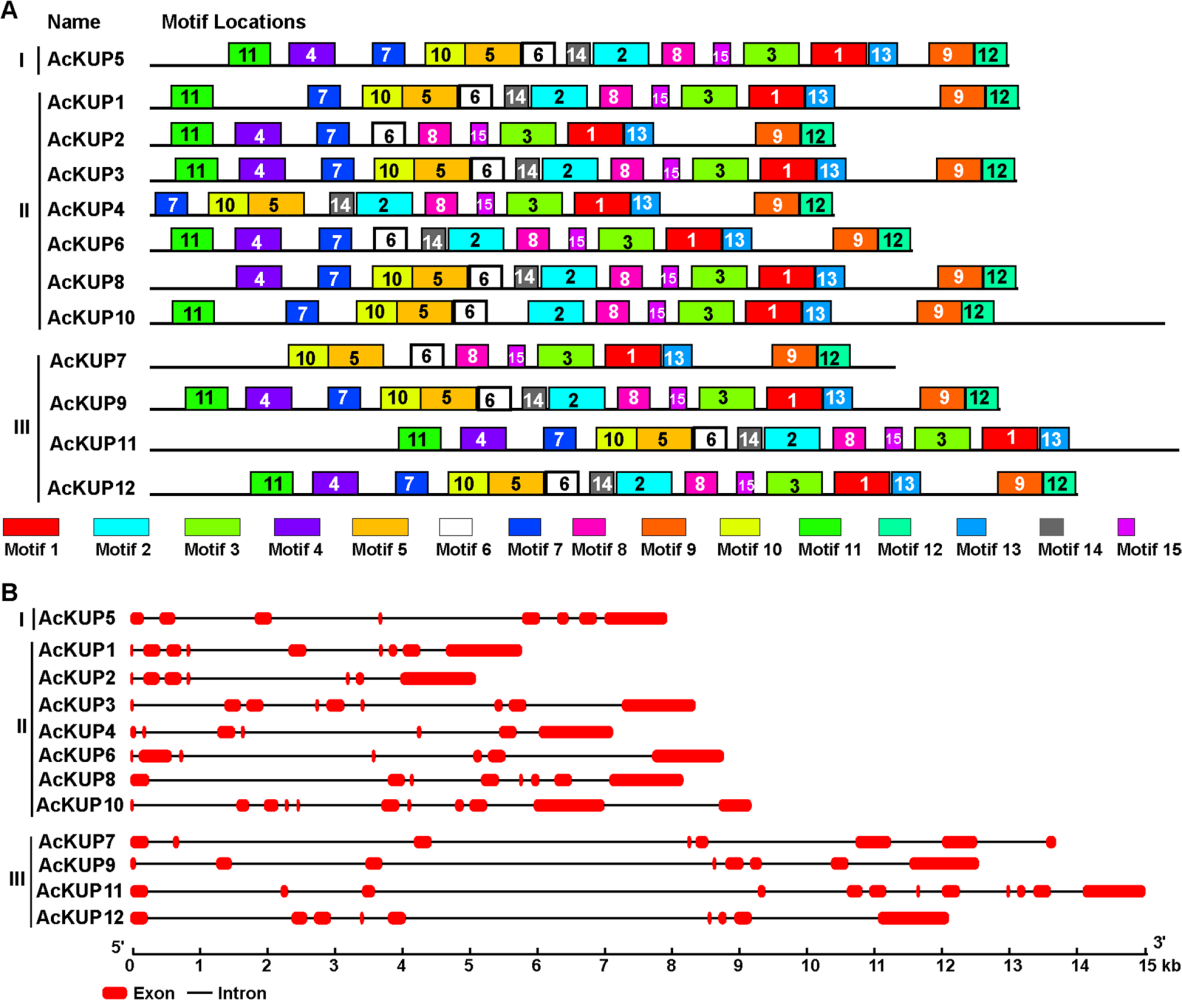
Conserved motif and gene structure analysis of the KT/HAK/KUP proteins in kiwifruit. **A** Conserved motif analysis of the KT/HAK/KUP proteins. Distribution of the conserved motifs was analyzed by MEME with 15 maximum number of motifs (http://meme-suite.org/). Each specific motif is marked by a different colored box. **B** Intron and exon structure of the KT/HAK/KUP proteins

To investigate their conserved domains, AcKUP proteins were submitted to MEME suite based on their evolutionary relationships, and 15 different motifs were identified (Fig. 2A). Motif 1, motif 3, motif 8, motif 13, and motif 15 were discovered in all the AcKUP proteins. The KUPs in Cluster I and III harbored motifs 1, 3, 5–6, 8–10, 12–13, and 15. Cluster II KUPs showed motifs 1, 3, 7–9, 12–13, and 15. Although some homologous KUPs had distinct motifs structures, such as AcKUP2/4 and AcKUP7/11, most of the homologous KUPs showed the same motif structure, including AcKUP1/3, AcKUP2/6, AcKUP8/10, AcKUP9/12. Together, these results indicate that each subgroup of AcKUPs shares similar motif features, further supporting the phylogenetic classification of KUP family. Gene structure analysis results showed that the *AcKUP* genes possessed 7 to 12 exons (Fig. 2B). Additionally, some *AcKUP* genes in the same cluster had the same number of exons, such as *AcKUP2*, *AcKUP3*, and *AcKUP6* in Cluster II, and *AcKUP7*, *AcKUP7* in Cluster III.

**Fig.3:**
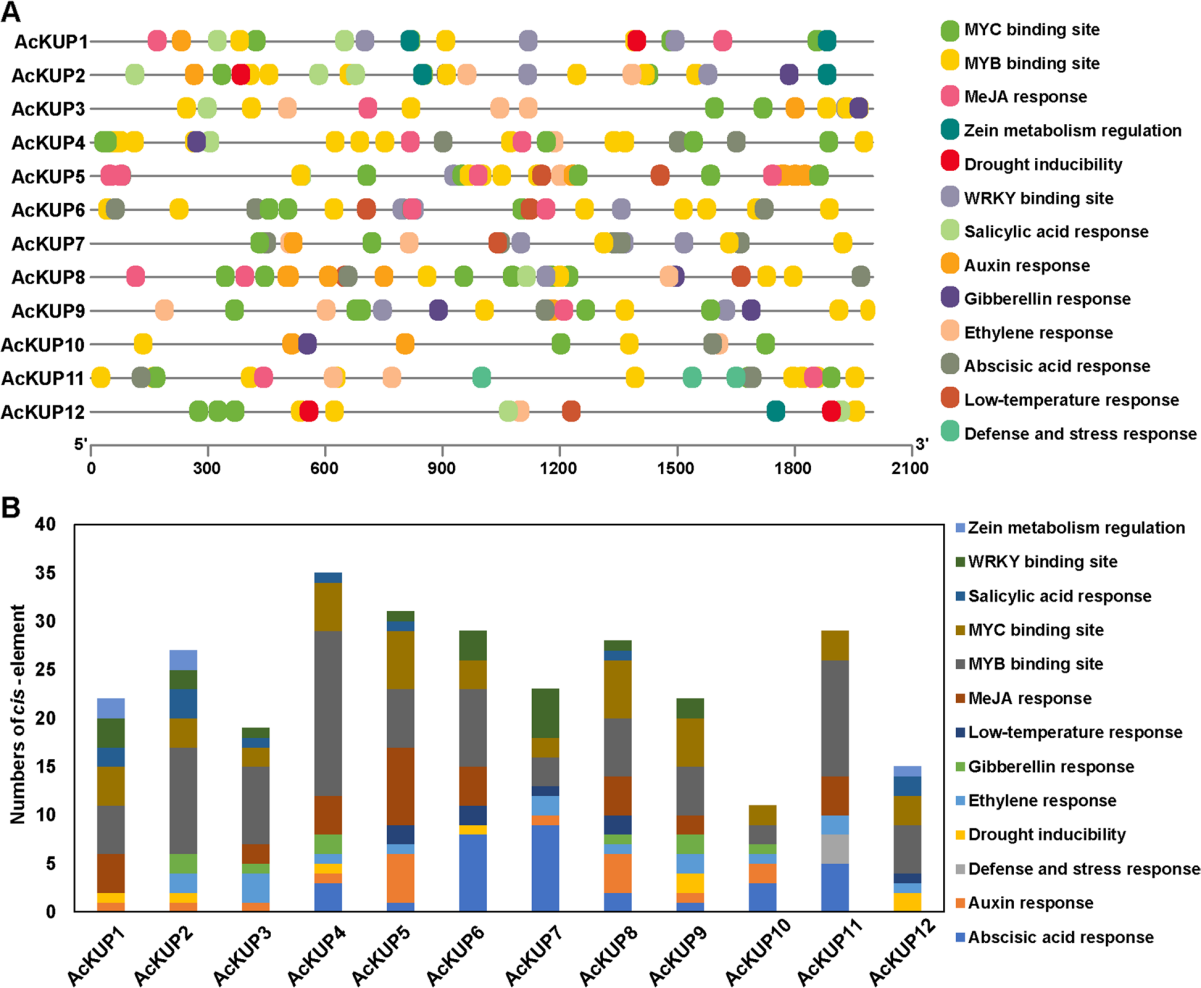
*Cis*-elements in the promoters of KT/HAK/KUP genes. **A** Distribution of *cis*-elements on the promoter of KT/HAK/KUP genes. **B** Number of *cis*-acting elements in the promoter of each KT/HAK/KUP genes

To excavate the potential function of *AcKUP* genes, *cis*-elements were predicted using PlantCARE. Stress responsive *cis*-elements, including ABA-responsive element (ABRE), dehydration-responsive element (DRE), low temperature-responsive element (LTRE), ethylene-responsive element (ERE), MYB-binding site (MBS), and gibberellin responsive element (GARE) in the *AcKUP* gene promoters were analyzed. To analyse the upstream promoter *cis*-elements, ~ 2,000 bp upstream sequences of coding sequence from *AcKUP* genes were isolated and identified using PlantCARE. The *cis*-elements related to hormones (such as ethylene, gibberellin, auxin, and salicylic acid) and light (G-box) were relatively abundant. MYB binding site (MBS) with a core sequence (CAACTG) were also identified (Fig. 3).

### Expression analysis of *AcKUP* genes

**Fig. 4:**
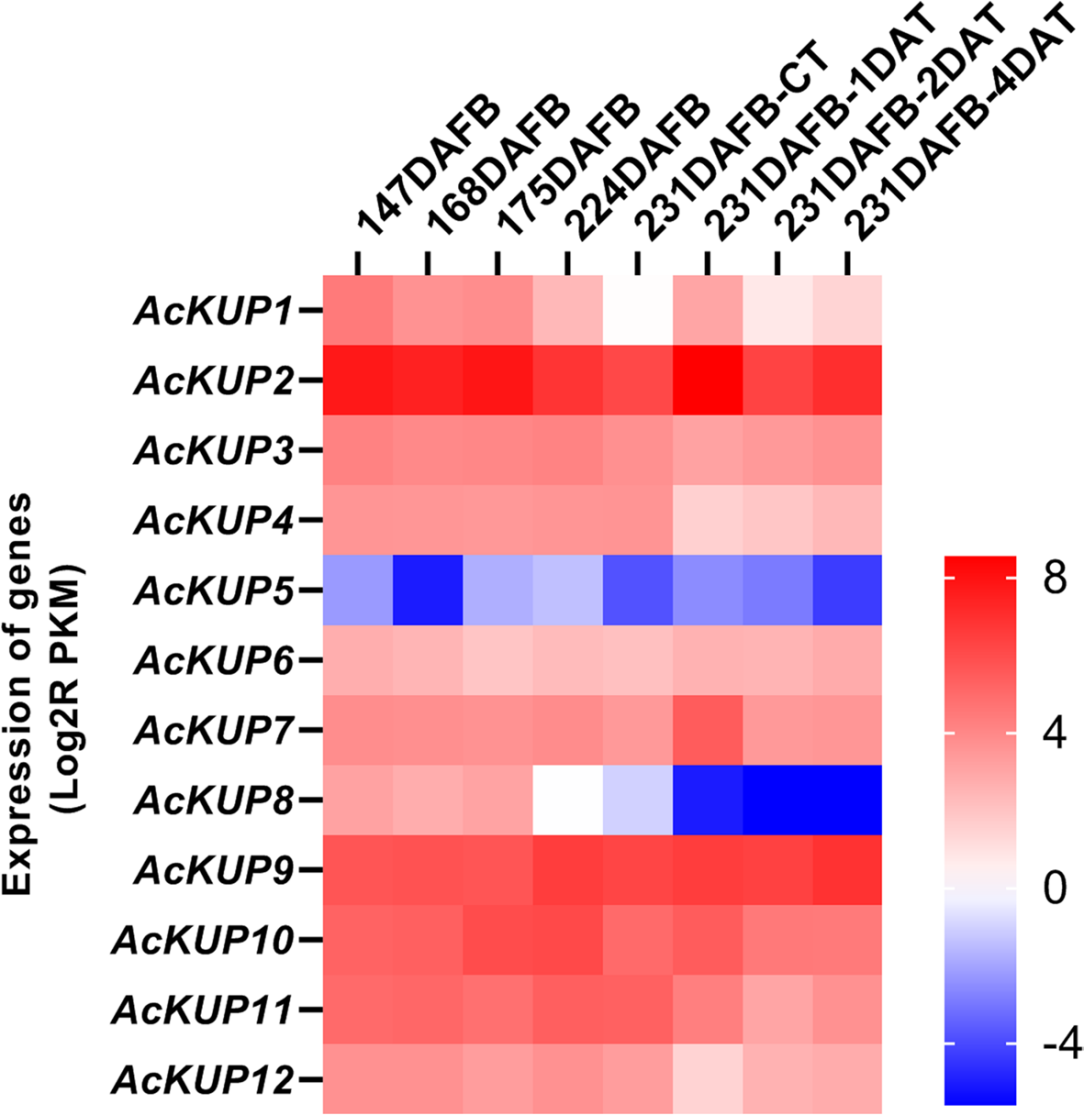
Expression profiles of *AcKUP* genes in different kiwifruit developmental stages. Red and blue boxes indicate high and low expression levels for each gene, respectively. DAFB, days after full bloom; DAT, day after treatment with ethylene

To understand the potential function of *AcKUP* genes in kiwifruit development and ripening, the transcript expression patterns of 12 *AcKUP* genes were investigated during fruit developmental stages using the expression profiles from the RNA-seq bioproject (PRJNA277383) of the KGD. As shown in Fig. 4, *AcKUP2* showed relatively high expression levels during the development and ripening process of kiwifruit.

**Fig. 5:**
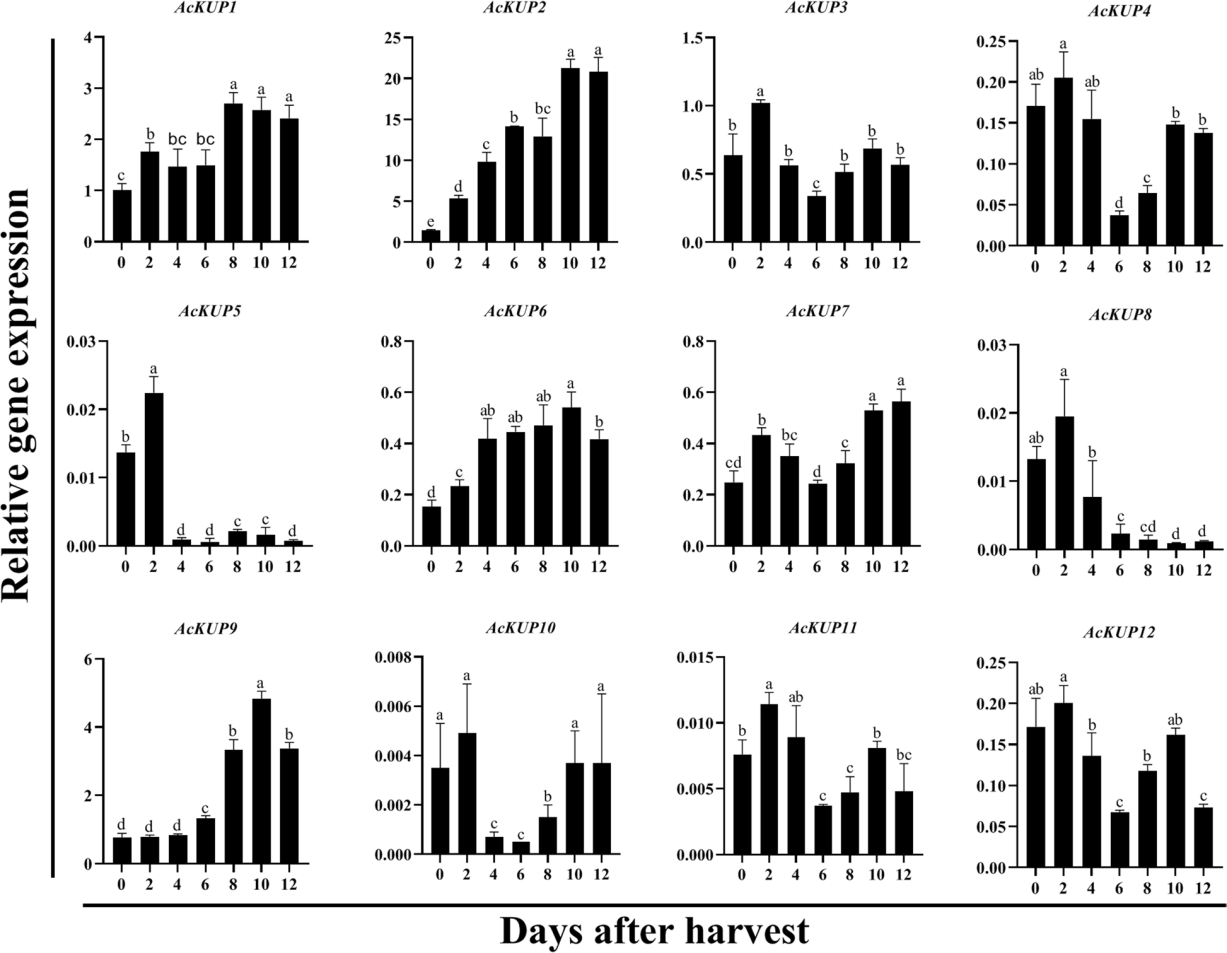
Relative expression analysis of 12 *AcKT/HAK/KUP* genes in kiwifruit seven postharvest stages. X-axis represents the days after postharvest. Y-axis represents the relative gene expression. Expression of *Actin* was used as internal control and to normalise the expression of *AcKT/HAK/KUP* genes. All values in the figure are relative to *AcKUP1* value on day zero after harvest. Error bars show the standard error among the three replicates. Bars with the same letter are not significantly different at *P* > 0.01, according to Student’s *t* test

To further explore whether *AcKUP* genes were involved in kiwifruit ripening, the expression pattern of *AcKUP* genes over the postharvest ripening stages was determined using qRT-PCR. The results showed that *AcKUP2* were the main expression members, exhibiting ripening-associated expression, and their expression increased significantly during kiwifruit postharvest ripening stages. The expression of *AcKUP9* was stable in the early stage after harvest but began to be up-regulated in the late stage. The other members expressed relatively low in the kiwifruit postharvest stages (Fig. 5).

**Fig. 6:**
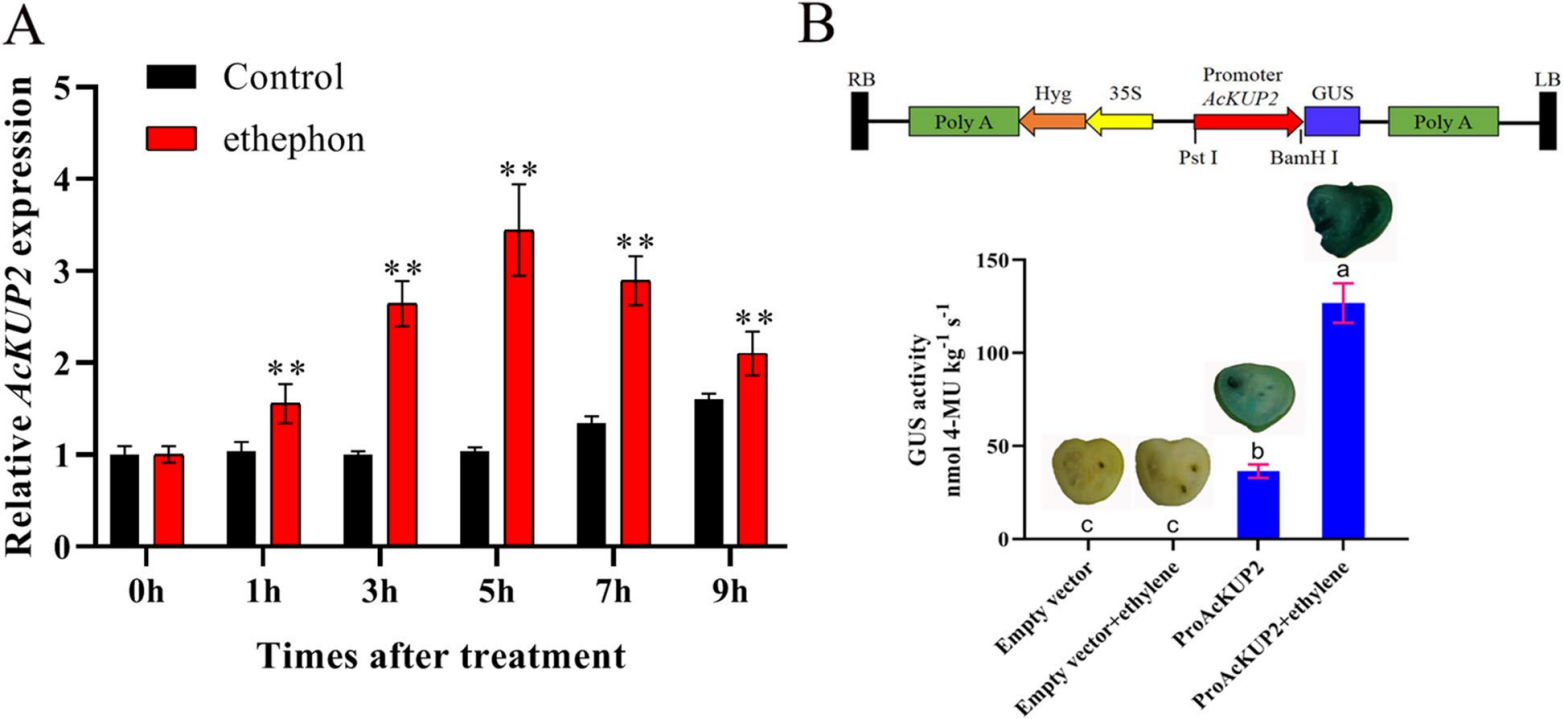
*AcKUP2* gene expression and promoter activity analysis. **A** The time course of ethephon-induced expression of *AcKUP2* gene. Kiwifruit-flesh cubes (1 cm^3^) were prepared from the fruit pulp and immediately immersed in 200 mL of equilibration solution (ethephon-treated: 50 mM ethephon, water-treated: control). Freshly cut kiwifruit discs were washed by gently stirring for 0, 1, 3, 5, 7, and 9 h in the equilibration solution, and gene expression was analysed by qRT-PCR. The data are expressed as mean ± SD. The asterisks indicate a significant difference according to student’s t test (**P < 0.01). **B** Schematic representation of *AcKUP2* promoter cloned in pCAMBIA1391 vector (promoterless vector) at Pst I and BamH I sites for measuring GUS activity and field-infiltration. **B** Inflorescence stem with GUS histochemical staining and GUS activity in tomato. Each date represents the mean of at least three replications and values are represented as mean ± SD. Bars with the same letter are not significantly different at *P* < 0.01

We, then, examined the effect of exogenous ethylene on the expression of *AcKUP2* and *AcKUP9* genes, and the results revealed that ethylene could significantly induce the expression of *AcKUP2* (Fig. 6A), but had no significant effect on the expression of *AcKUP9* (Fig. S1), which was consistent with the result of the RNA-seq.

### Subcellular localisation of AcKUP2

**Fig. 7:**
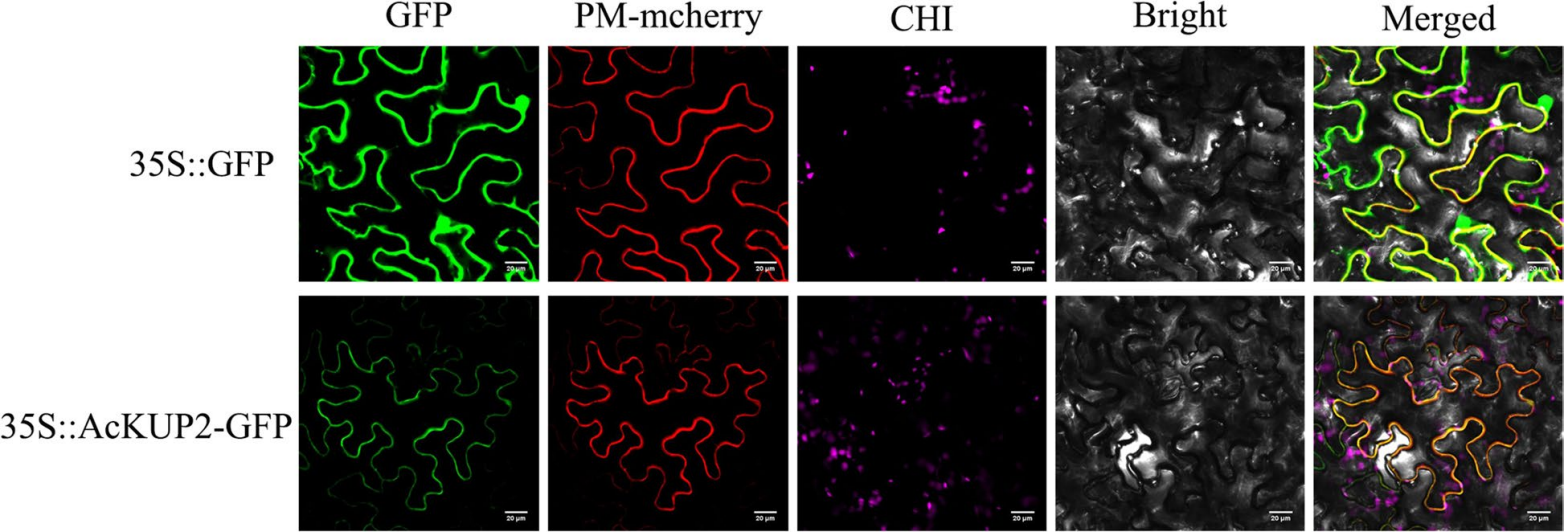
Subcellular localisation of AcKUP2. Subcellular localisation of GFP and AcKUP2-GFP driven by the CaMV35S promoter expressed in tobacco epidermal cells

AcKUP2 were expected to located in the plasma membrane. We used confocal microscopy to examine the expression and subcellular localisation of AcKUP2–GFP fusion protein in the epidermal cells of tobacco. Fluorescence microscopy showed that the AcKUP2–GFP fusion protein was distributed only within the plasma membrane of the tobacco epidermal cells. This result contrasted with the observation for the GFP control, which showed fluorescence throughout the tobacco epidermal cells (Fig. 7).

### Analysis of *AcKUP2* promoter activity

The *cis*-elements related to hormones were relatively abundant in the promoter of AcKUP2 gene (Fig. 3; Table S2). These results suggest the possible transcriptional regulation of the *AcKUP2* gene. Ethylene is essential for the postharvest ripening of kiwifruit, so we determined the effect of ethylene on the promoter activity of *AcKUP2*. Fusion construct pro*AcKUP2*–GUS was transiently expressed in tomato fruit and used to check the activities of the promoter. Histochemical staining and GUS activities revealed that GUS activity increased strikingly with ethylene treatment (Fig. 6B). These results showed that *AcKUP2* is directly induced in response to ethylene, which might be closely related to fruit ripening.

### AcERF15 binds to *AcKUP2* promoter

**Fig. 8:**
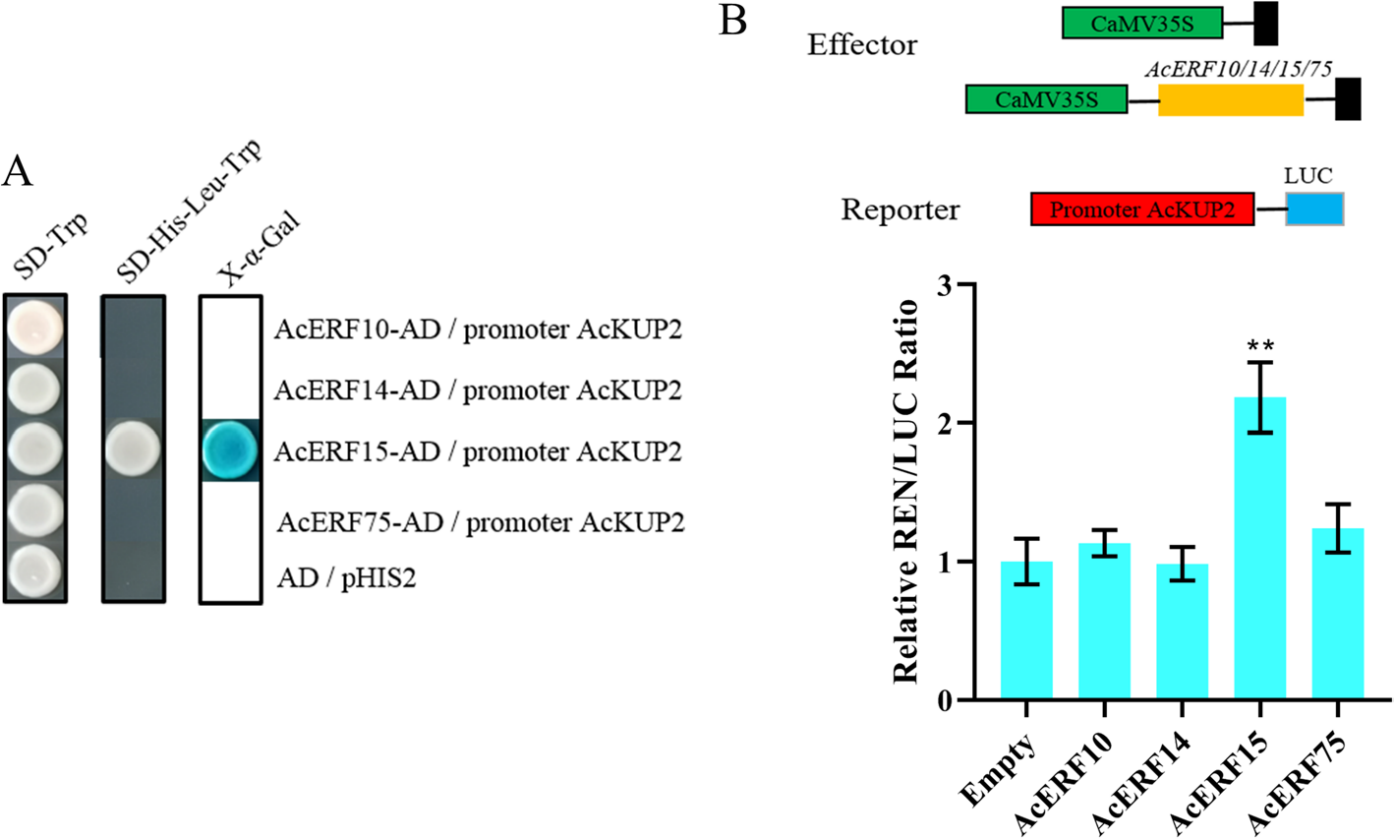
AcERF15 enhanced the activity of *AcKUP2* promoter. **A** The growth status of yeasts on three different types of media (SD–Trp, SD–His–Leu–Trp, and SD–His–Leu–Trp + X–α–Gal) after the yeasts were transformed with a combination of effector and reporter vectors is shown. **B** The CDS of *AcERFs* were cloned into the pGreenII 0029 62–SK vector driven by the 35S promoter as an effector, and the promoter sequence of *AcKUP2* was inserted into the pGreenII 0800–LUC vector as reporter. Dual-luciferase assays in *Nicotiana benthamiana* leaves were performed to analyse the activity enhancement of *AcKUP2* promoter by AcERFs. An empty vector was used as the reference control. “**” indicates significant differences at *P* < 0.01

At present, 119 ERF family members have been isolated and identified from the kiwifruit genome; among these members, ERF10/14/15/75 are considered to be putative activators for kiwifruit ripening and softening [33]. Because the activity of *AcKUP2* promoter could be enhanced by ethylene treatment, we investigated whether AcERFs regulate the expression of *AcKUP2* during fruit ripening. First, we performed a Y1H experiment. The CDS of *AcERF10/14/15/75* were cloned into the pGADT7 vector for the effector construct, and the *AcKUP2* promoter fragment was cloned into the pHIS2 vector for the reporter construct. The yeast cells co-transformed with AcERF15 and *AcKUP2* promoter grew well, whereas cells co-transformed with another vector did not (Fig. 8A). To further determine whether AcERFs could enhance *AcKUP2*-promoter activity, we performed transient expression assays in tobacco leaves using dual-luciferase reporters. The results showed that the interaction of AcERF15 with the *AcKUP2* promoter led to a nearly two-fold increase in the relative LUC/REN ratio (Fig. 8B). These results suggest that AcERF15 enhances the transcription of *AcKUP2* by directly binding to its promoter.

## Discussion

Mineral elements play an important role in fruit development and maturation. The softening of the fruit during ripening is accompanied by a decrease in calcium content [34]. Mg^2+^ is the core component of chloroplast. From the fruit expansion to the colouring stage, the demand for magnesium continues to increase. Magnesium deficiency will affect the quality of the fruit [35]. Nevertheless, potassium is the most abundant mineral element in kiwifruit, and its content is always maintained at a high level during fruit ripening. Similarly, K content remains high in some other fleshy fruits, such as banana [9], strawberry [28], and passion fruit [36]. In our study, K content kept increasing during fruit softening and decreases after that (Table 1). This result is similar to the pattern of changes in K content in bananas [9].

KT/HAK/KUP is the largest K^+^ transporter family in plants, primarily responsible for K^+^ uptake and transport; it plays an important role in plant growth, development, osmotic potential regulation, and stress resistance [37]. The K^+^ transporters *KT/HAK/KUP* are widely present in different plant species. Currently, *A. thaliana*, rice, maize, and peach have been described to contain 13 [38], 25 [24], 27, [39], and 17 [31] members of the *KT/HAK/KUP* family, respectively. In this study, 12 members of the *KT/HAK/KUP* family, named *AcKUP1–AcKUP12*, were identified by the genome identification of kiwifruit. According to gene sequence homology analysis and phylogenetic tree construction, KT/HAK/KUP was divided into four gene evolutionary groups [38]. There is only one member of the *A. thaliana* KT/HAK/KUP family distributed in group I, and the other members are mainly distributed in groups II and III [40]; the same was found in kiwifruit. Many members of group I, such as AtHAK5, OsHAK1, OsHAK5, and ThHAK5, can respond to low-K stress and significantly improve the high-affinity absorption of K by yeast and *E. coli*. [27, 41]. The sequence and function of group II members are quite different. For example, AtKUP1 can mediate both high- and low-affinity K^+^ transport [22], whereas CnHAK1 only acts as a low-affinity K + transporter [42]. Members of the KT/HAK/KUP transporter family are located on the membranes of different plant organelles [43]. Family members of the same group may have different subcellular localisation and may perform different cell biological functions. For example, group II members OsHAK2 and OsHAK3 are located on the plasma membrane, while OsHAK10 is located on the vacuole membrane [41]. In our study, AcKUP2, a member of group II, was located on the plasma membrane, thereby verifying its K^+^ transporter activity.

At present, the expression of *KT/HAK/KUP* genes has been analysed in many fleshy fruits, and it was found that this gene family may play an important role in fruit development and ripening. Both *VvKUP1* and *VvKUP2* have the ability to transport K^+^ and participate in fruit development and ripening by regulating K^+^ transport [4]. *PpeKUP1* and *PpeKUP2* may be the major transporters that function in the K^+^ accumulation and homeostasis in the fruit skin, which were closely involved in peach fruit development [31]. *SlHAK10* was strongly expressed in tomato fruits than in other tissues [25]. Most *PbrKT/HAK/KUP* genes were expressed during the development of pear fruits, indicating these genes play an important role in the process of fruit ripening [26]. In this study, *AcKUP1, AcKUP2*, and *AcKUP9* were abundantly expressed in kiwifruit during postharvest ripening, and the expression levels of *AcKUP2* and *AcKUP9* increased with fruit ripening and softening. Therefore, *AcKUP2* and *AcKUP9* may co-regulate K content during postharvest ripening of kiwifruit.

Gene expression at various stages of plant growth and development is regulated by various hormonal signals, and K^+^ transporters are no exception [44]. The expression of *OsHAK1*, *OsHAK7* and *OsHAK10* were regulated by naphthylacetic acid, gibberellin, and kinetin [24]. Ethylene can improve the tolerance of *A. thaliana u*nder low-K^+^ stress. It is speculated that ethylene, as a component of the low-K signalling pathway, can directly act on K^+^ transporters or regulate the expression of K^+^ transporter-related genes by stimulating ROS production, and ultimately increase K^+^ uptake in plants [45]. Under water stress, ABA can up-regulate the expression of *KUP6*. The inactivation of *KUP6* and its homologs *KUP2* and *KUP8* will affect the stomata closure mediated by ABA and the response of plants to drought stress [46]. In addition, auxin can promote the uptake of K^+^ in plants by regulating the K^+^/H^+^ co-transport activity of *OsHAK5* [27]. In our study, there were many *cis*-acting elements response to phytohormones (ethylene, gibberellin, salicylic acid, auxin) in *AcKUP2* promoter, and the activity of *AcKUP2* promoter was obviously induced by exogenous ethylene (Fig. 6). The expression of *KT/HAK/KUP* is also regulated by some transcription factors. The overexpression of *DDF2*, *JLO*, *TFII-A* and *bHLH121* can activate *HAK5* and enhance the response of *A. thaliana* to low-K^+^ and salt stress [47]. RAP2.11 was identified bound to a GCC-box of the *AtHAK5* promoter regulating *AtHAK5* expression under low-K^+^ conditions; its overexpression could up-regulate the expression of a large number of genes involved in ethylene and calcium signalling and reactive oxygen species production [48]. When K is sufficient, ARF2 can directly bind to the AuxREs motif of the *HAK5* promoter to inhibit the expression of *HAK5* [49]. Our study demonstrates that AcERF15, an ethylene response factor, can directly bind to the *AcKUP2* promoter to stimulate its expression (Fig. 8). Therefore, we suggest that ethylene regulates the expression of *AcKUP2* through AcERF15, thereby participating in the postharvest ripening process of kiwifruit.

## Methods

### Fruit firmness and total soluble solids (TSS)

‘Hongyang’ kiwifruit fruits (*Actinidia chinensis* Planch.) were obtained in September 2019 at the commercial mature stage (142 days after pollination, TSS of 6.5–7.0%) from a commercial orchard under unified management in Fengxin County, Jiangxi Province, China (28.7° N, 115.38° E, and elevation 65 m). Fruit firmness and TSS were measured as described in a previous study [50] using a fruit-texture analyser (TMS-Touch, FTC, Sterling, VA, USA) and a refractometer (PL-1, Atago Co. Ltd., Tokyo, Japan), respectively; 20 single-fruit replicates were used per test. The fruits were, then, used for pulp-sample collection (without skins or seeds) at 0, 2, 4, 6, 8, 10, and 12 d. In the sequence, the pulp samples were frozen in liquid nitrogen and stored at -80 °C until use.

### Measurement of mineral concentrations

For mineral analysis, approximately 2.0 g dried pulp and 30 mL nitric–perchloric (4:1, v/v) digestive solution was thoroughly mixed and left to stand for 4 h. After digestion, 8 mL of 50% nitric acid was added to the mixture, and the volume was adjusted to 50 mL with distilled water. The mineral concentrations were measured as described previously [36]. A blank control was used for the analysis. Each experiment was repeated three times.

### Sequence identification, gene structure, conserved motif, and phylogenetic analysis of *KT/HAK/KUP* genes

Candidate genes encoding *KT/HAK/KUP* were retrieved by BLASTP search against the KGD (http://kiwifruitgenome.org/) [51], using *A. thaliana* KT/HAK/KUP proteins as queries. The length, molecular weight (MW), and theoretical isoelectric point (pI) of KT/HAK/KUP proteins were calculated using the ProtParam tool (https://web.expasy.org/protparam/) [52]. Intron/exon structure analysis was performed using the Gene Structure Display Server (http://gsds.cbi.pku.edu.cn) [53]. CDS and genomic sequences of *KT/HAK/KUP* genes were submitted to obtain the gene structure and draw diagram. The distribution of conserved motifs of KT/HAK/KUP in kiwifruits was analysed using the MEME suite 5.4.1 (http://meme-suite.org/) [54, 55] with 15 maximum numbers of motifs. Phylogenetic analysis was conducted using software MEGA 7.0 [56]. Evolutionary history was inferred using the neighbour-joining method with 1,000 replicates.

### Gene expression analysis

Total RNA was isolated using a Quick-RNA™ isolation kit (Huayueyang, Beijing, China). Residual DNA in the isolated RNA was digested by incubating the sample with DNase I (Huayueyang, Beijing, China). RNA concentrations were measured using a NanoDrop spectrophotometer (Thermo Fisher Scientific, Waltham, MA, USA). RNA (1 μg) was used for cDNA synthesis using the Hifair® II 1st strand cDNA synthesis kit (Yeasen, Shanghai, China). A TB Green™-based qRT-PCR was performed using a CFX96 Touch Real-time PCR detection system (Bio-Rad, Hercules, CA, USA). The qRT-PCR was conducted using a 20 μL reaction mixture containing 2 μL of template cDNA, 0.1 μM of each of the two gene-specific primers (Table S1), and 10 μL of 2 × TB® Green Master Mix (Takara, Dalian, China). The amplification programme consisted of one cycle of 1 min at 95 °C, 40 cycles of 15 s at 95 °C, and 25 s at 63 °C. Fluorescence was measured with a 55–95 °C-melting-point curve. Kiwifruit *Actin* was used as an internal control [50]. Differences in the cycle threshold between target and *Actin* genes were used to estimate the relative transcription level of the target gene. Three biological replicates and three technical replicates were included to ensure the accuracy of the expression data.

### Subcellular localisation

Subcellular locations of KT/HAK/KUP proteins were predicted using the Euk-mPLoc 2.0 server (http://www.csbio.sjtu.edu.cn/bioinf/euk-multi-2/) [57]. The *AcKUP2* ORF without a termination codon was further inserted into a super 1300 vector to generate the *AcKUP2–GFP* construct. *Agrobacterium tumefaciens*, using *AcKUP2–GFP* and *CaMV35S–GFP* vectors (1:1 ratio), was, then, transient transformed. Fully expanded leaves of tobacco (*Nicotiana tabacum* L. ‘USA’) plants were agro-infiltrated using 0.5 mL of bacterial suspension in a 1-mL syringe into the abaxial surface of the intact leaf. After 3 d, GFP fluorescence was visualised using confocal microscopy. The wavelength used in detecting GFP and mCherry fluorescence were 488 nm and 552 nm, respectively.

### Promoter activity assay of *AcKUP2*

A sequence of 2000 bp upstream from the start codon of each *KT/HAK/KUP* gene was downloaded from kiwifruit genome. Then *cis*-elements in promoter of each *KT/HAK/KUP* gene were predicted by using the PlantCARE server (http://bioinformatics.psb.ugent.be/webtools/plantcare/html/) [58] and with Dual Synteny Plotter software (https://github.com/CJ-Chen/TBtools) [59].

The putative promoter region of *AcKUP2*, a 1,710-bp PCR fragment upstream of the start codon ATG was further amplified. The PCR product was digested with *Pst* I, and the *Bam*H I sequence was cloned in front of the *GUS* gene in the pCAMBIA1391 vector (promoterless vector), yielding the construct proAcKUP2–GUS. Then, *A. tumefaciens* containing the proAcKUP2-GUS or control vector was injected into a tomato fruit at the breaker stage until the whole fruit was infiltrated. After 3 d, agro-infiltrated fruit discs were soaked in petri dishes filled with 100 μM ethephon and incubated for 12 h at room temperature. GUS staining was performed using a GUS staining detection kit (Huayueyang, Beijing, China). The fruit discs were soaked in the GUS staining solution, and held at 37℃ for 1 h to overnight. After stained, the discs were decolourised with 70% ethanol for 3 times until the negative control was white. The blue dots that appeared on the white background were GUS expression sites.

### Yeast one-hybrid (Y1H) assay

The ORF of *AcERF10/14/15/75* were inserted into the pGADT7 vector, and the promoter of *AcKUP2* was cloned into a pHIS2 vector. The promoter *AcKUP2*–pHIS2 reporter vector and *AcERF10/14/15/75*–AD effector vector were transferred to the Y187 yeast strain. Yeast transformants were grown and selected on SD/–Trp or SD/–Trp/–Leu/–His media.

### Dual-luciferase reporter assay

For the measurement of the effects of AcERFs on the transcription of *AcKUP2*, the ORF of *AcERFs* were cloned into the pGreenII 0029 62–SK vector driven by the 35S promoter as an effector, and promoter sequences of *AcKUP2* were inserted into the pGreenII 0800–LUC vector as reporter. All the constructs were transformed in *A. tumefaciens* and, then, injected into tobacco leaves according to the method of subcellular localisation. LUC and REN luciferase activity were measured using a dual-luciferase assay kit (YEASEN, Shanghai, China) according to the manufacturer recommendation. The LUC to REN ratio was calculated. At least six biological replicates were performed per assay.

## Supplementary Information

**Additional file 1: Table S1.** *KT/HAK/KUP *transporter genes identified in kiwifruit and their sequence characteristics.

**Additional file 2: Table S2.** Main regulatory motifs in the *AcKUP2* promoter. **Table S3. **Primers for qRT-PCR. **Table S4. **Primers for vector construction.

**Additional file 3: Figure S1. **The time course of ethephon-induced expression of *AcKUP9* gene.

## Data Availability

Data generated or analyzed during this study are included in this article and its supplemental files. The RNA-Seq data (bioproject accession PRJNA277383, http://kiwifruitgenome.org/rnaseq/other/3) used and analyzed during this study is publicly available in the kiwifruit genome database.
