# Supplementary material for: Ethylene-induced potassium transporter AcKUP2 gene is involved in kiwifruit postharvest ripening
Source: BMC Plant Biol. 2022 Mar 9;22:108. doi: 10.1186/s12870-022-03498-9 (PMC8905847; doi:10.1186/s12870-022-03498-9)
Supplement: Supplementary file 2 — Additional file 2: Table S2. Main regulatory motifs in the AcKUP2 promoter. Table S3. Primers for qRT-PCR. Table S4. Primers for vector construction. [file 12870_2022_3498_MOESM2_ESM.docx]

**Table S2.** Main regulatory motifs in the *AcKUP2* promoter.

| *cis*-element | Sequence | Probable function |
| --- | --- | --- |
| CRT/DRE | GTCGGT | ERF (ethylene response factor) binding site |
| ERE | TTTGAAA | ERF (ethylene response factor) binding site |
| GARE-motif | TCTGTTG | Gibberellin-responsive element |
| P-box | CCTTTTG | Gibberellin-responsive element |
| MBS | CAACTG | MYB binding site |
| MBSI | TTTTTACGGTTA | MYB binding site |
| G-Box | GCCACGTGGA | Involved in light responsiveness |
| TCA-element | CCATCTTTTT | Salicylic acid-responsive element |
| TGA-element | AACGAC | Auxin-responsive element |

**Table S3.** Primers for qRT-PCR

| Name | Forward | Reverse |
| --- | --- | --- |
| *AcKUP1* | GTCCTACCGTTGTATTGTCCG | CCGTAGTTCCACGCCTGTGA |
| *AcKUP 2* | GAGTCCGAGTCTGAGTTTGA | CGAGAATGAATGCTGTCCC |
| *AcKUP3* | TTGGGTTTATTGAGGGTGTC | TTCTTGCGAGTTCCATAGTG |
| *AcKUP4* | CATCTGAGAAGTTCGGGACA | GATGACTTGCTATTGGGTGC |
| *AcKUP5* | GAAGAAAGGGTTGTTTGGGTA | TTATTGGAGCGAAGGTGTAG |
| *AcKUP6* | TGCTGGTCACTACCTGCCTAA | CCACGCTCCTTCAAGAAACT |
| *AcKUP7* | CCTGTTTCTGTAGTGCCTCA | AAGCTGCTCAAATGTCTGGT |
| *AcKUP8* | TTGGCGTAGTGTTTACTGA | CTAGAGGCACATAAGGGAC |
| *AcKUP9* | GACCAGCCTATGGATGAAGAAG | ACAAAGGAGATGTCCCTAAGTC |
| *AcKUP10* | AGATAGGGAGTTCAATCCACG | AGTCGGTCCACAAGTTAAGCA |
| *AcKUP11* | AATACCAGAGCGAGGTCAGCG | AATAGCCGGAAGGTCCAGCAT |
| *AcKUP12* | AAAGCACCACTGACATAGCG | AATCTCCACCGTCCCAAATA |
| *AcActin* | TGGAATGGAAGCTGCAGGA | CACCACTGAGCACAATGTTGC |

**Table S4** Primers for vector construction.

| **Name** | **Forward** | **Reverse** |
| --- | --- | --- |
| **Subcellular localization** | | |
| AcKUP2–GFP | CCAAATCGACTCTAGTCTAGAATGGATCCGGACTACTCGCG | GCCCTTGCTCACCATGGTACCCACAACATAGACCATACCAACCTC |
| **Promoter activity** | | |
| proAcKUP2–GUS | TGGGCCCGGCGCGCCAAGCTTTGGGTTTCTTCGTCCGTTCA | TCTTAGAATTCCCGGGGATCCGCTTCTCAGGCCTTAATTTTCA |
| **Y1H** | | |
| AcERF10-AD | CGTACCAGATTACGCTCATATGATGAATTCCCAATTCACCCAA | GCAGCTCGAGCTCGATGGATCCCTAAGATGAATTTAGTAGCTCCTC |
| AcERF14-AD | CGTACCAGATTACGCTCATATGATGGCGATTGATCTTGATCT | GCAGCTCGAGCTCGATGGATCCTCACCATGGGTTGTCAGTAC |
| AcERF15-AD | CGTACCAGATTACGCTCATATGATGCAAGAAAACAACAACCC | GCAGCTCGAGCTCGATGGATCCCTAATTTGCAAGAACTTCCC |
| AcERF75-AD | CGTACCAGATTACGCTCATATGATGTGCGGCGGTTCAATCCT | GCAGCTCGAGCTCGATGGATCCTCAGGCAAACGGAGGAGGAA |
| proAcKUP2–pHIS2 | GGGCGAATTCCCGGGGAGCTCTGGGTTTCTTCGTCCGTTCA | CGGATCGATTCGCGAACGCGTGCTTCTCAGGCCTTAATTTTCA |
| **Dual-luciferase** | | |
| AcERF10-62 SK | GCGGCCGCTCTAGAACTAGTATGAATTCCCAATTCACCCAA | TCGAATTCCTGCAGCCCGGGCTAAGATGAATTTAGTAGCTCCTC |
| AcERF14-62 SK | GCGGCCGCTCTAGAACTAGTATGGCGATTGATCTTGATCT | TCGAATTCCTGCAGCCCGGGTCACCATGGGTTGTCAGTAC |
| AcERF15-62 SK | GCGGCCGCTCTAGAACTAGTATGCAAGAAAACAACAACCC | TCGAATTCCTGCAGCCCGGGCTAATTTGCAAGAACTTCCC |
| AcERF75-62 SK | GCGGCCGCTCTAGAACTAGTATGTGCGGCGGTTCAATCCT | TCGAATTCCTGCAGCCCGGGTCAGGCAAACGGAGGAGGAA |
| proAcKUP2–0800 | TCGAATTCCTGCAGCCCGGGTGGGTTTCTTCGTCCGTTCA | GCGGCCGCTCTAGAACTAGTGCTTCTCAGGCCTTAATTTTCA |
