# Supplementary material for: Ethylene-induced potassium transporter AcKUP2 gene is involved in kiwifruit postharvest ripening
Source: BMC Plant Biol. 2022 Mar 9;22:108. doi: 10.1186/s12870-022-03498-9 (PMC8905847; doi:10.1186/s12870-022-03498-9)
Supplement: Supplementary file 3 — Additional file 3: Figure S1. The time course of ethephon-induced expression of AcKUP9 gene. [file 12870_2022_3498_MOESM3_ESM.docx]

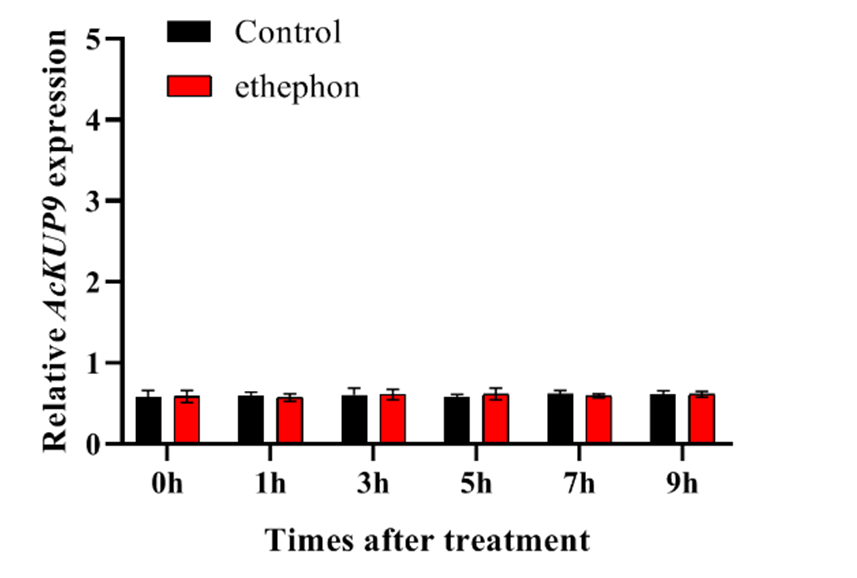


**Fig. S1** The time course of ethephon-induced expression of *AcKUP9* gene. Kiwifruit-flesh cubes (1 cm^3^) were prepared from the fruit pulp and immediately immersed in 200 mL of equilibration solution (ethephon-treated: 50 mM ethephon, water-treated: control). Freshly cut kiwifruit discs were washed by gently stirring for 0, 1, 3, 5, 7, and 9 h in the equilibration solution, and gene expression was analysed by qRT-PCR. The data are expressed as mean ± SD. The asterisks indicate a significant difference according to student’s t test (***P* < 0.01).
